# BAG5 Promotes Alpha-Synuclein Oligomer Formation and Functionally Interacts With the Autophagy Adaptor Protein p62

**DOI:** 10.3389/fcell.2020.00716

**Published:** 2020-08-04

**Authors:** Erik L. Friesen, Yu Tong Zhang, Rebecca Earnshaw, Mitch L. De Snoo, Darren M. O’Hara, Victoria Agapova, Hien Chau, Sophie Ngana, Kevin S. Chen, Lorraine V. Kalia, Suneil K. Kalia

**Affiliations:** ^1^Department of Laboratory Medicine and Pathobiology, University of Toronto, Toronto, ON, Canada; ^2^Division of Genetics and Development, Krembil Research Institute, Toronto, ON, Canada; ^3^Division of Neurology, Department of Medicine, University of Toronto, Toronto, ON, Canada; ^4^Division of Neurosurgery, Department of Surgery, University of Toronto, Toronto, ON, Canada

**Keywords:** alpha-synuclein, chaperones, bcl-2 associated athanogene, BAG5, proteostasis, p62, sequestosome-1

## Abstract

Molecular chaperones are critical to maintaining intracellular proteostasis and have been shown to have a protective role against alpha-synuclein-mediated toxicity. Co-chaperone proteins regulate the activity of molecular chaperones and connect the chaperone network to protein degradation and cell death pathways. Bcl-2 associated athanogene 5 (BAG5) is a co-chaperone that modulates proteostasis by inhibiting the activity of Heat shock protein 70 (Hsp70) and several E3 ubiquitin ligases, resulting in enhanced neurodegeneration in models of Parkinson’s disease (PD). Here we identify a novel interaction between BAG5 and p62/sequestosome-1 (SQSTM1), suggesting that BAG5 may bridge the chaperone network to autophagy-mediated protein degradation. We found that BAG5 enhanced the formation of pathogenic alpha-synuclein oligomers and regulated the levels and subcellular distribution of p62. These results extend the role of BAG5 in alpha-synuclein processing and intracellular proteostasis.

## Introduction

Parkinson’s disease (PD) is an incurable neurodegenerative disease which affects 1–2% of the population over the age of 60 (Kalia and Lang, 2015). PD is characterized by a significant loss of dopaminergic neurons within the substantia nigra pars compacta as well as the presence of Lewy bodies (LBs), intracellular inclusions comprised largely of aggregated alpha-synuclein (Kalia et al., 2013). While the exact mechanisms are still unknown, oligomeric species of alpha-synuclein are strongly believed to contribute to the cell death observed in PD and other diseases associated with LBs including dementia with LBs, multiple system atrophy, and Alzheimer’s disease (Kim et al., 2014).

Various factors modulate alpha-synuclein processing and aggregation including molecular chaperones. Chaperones serve to fold nascent proteins, refold misfolded proteins, or direct misfolded proteins for degradation via either the ubiquitin-proteasome system (UPS) or autophagy lysosome pathway (ALP) (Friesen et al., 2017). Heat shock protein 70 (Hsp70) is a chaperone which has been shown to be involved in alpha-synuclein processing and preferentially binds to alpha-synuclein fibrils (Aprile et al., 2017). Hsp70 can reduce levels of misfolded and aggregated alpha-synuclein, and protect against alpha-synuclein-mediated toxicity (Auluck et al., 2002; Klucken et al., 2004; Dedmon et al., 2005; Flower et al., 2005; Huang et al., 2006).

Co-chaperones are proteins that regulate the function of chaperones by modulating their ATPase activity. One such family of co-chaperones is the BAG family of co-chaperones which act as nucleotide exchange factors that promote ADP release (Höhfeld and Jentsch, 1997; Arakawa et al., 2010; Rampelt et al., 2012; Friesen et al., 2017). The bcl-2 associated athanogene (BAG) co-chaperone family includes six members that are defined by the presence of a C-terminal BAG domain (Kabbage and Dickman, 2008). The BAG domain consists of three amphipathic alpha helices and ranges from 74 to 112 amino acids in size (Arakawa et al., 2010). This domain is responsible for a physical association with Hsp70, dimerization with other BAG proteins (Takayama et al., 1997), and other interactions critical for cell function. BAG co-chaperones not only regulate Hsp70 folding activity but also interact with proteins within the UPS and ALP, which together facilitate the degradation of Hsp70 client proteins (McDonough and Patterson, 2003; Xilouri and Stefanis, 2015), and function in other processes including cell division and apoptosis (Kalia et al., 2010; Friesen et al., 2017). For example, BAG1 contains a ubiquitin-like domain, which interacts with the 26S proteasome and thereby facilitates a physical link between Hsp70 and the UPS (Lüders et al., 2000). BAG1 is also a Bcl-2 interacting protein with anti-apoptotic activity (Takayama et al., 1995). BAG3 interacts with p62 [or sequestosome-1 (SQSTM1)], an ALP “adaptor” protein, which promotes proteostasis by facilitating increased protein degradation via the ALP as cells age (Gamerdinger et al., 2009).

BAG5 is unique among the BAG co-chaperones in that it contains five BAG domains rather than one. BAG5 interacts with Hsp70 and inhibits its folding activity (Kalia et al., 2004). BAG5 also interacts with and inhibits the ubiquitin E3 ligase activities of parkin and C-terminal Hsp70 interacting protein (CHIP) (Kalia et al., 2004, 2011). The inhibition of Hsp70, parkin, and CHIP by BAG5 disrupts proteostasis, mitophagy (De Snoo et al., 2019), and promotes the formation of alpha-synuclein oligomers, as well as other protein aggregates, which contribute to neuronal death (Kalia et al., 2013). Consistent with these findings, BAG5 was found to promote dopaminergic neuron death in the substantia nigra in rodent models of PD (Kalia et al., 2004) and interact with other PD relevant proteins including LRRK2, PINK1, and DJ-1 (Beilina et al., 2014; Wang et al., 2014; Qin et al., 2017; Tan et al., 2019; De Snoo et al., 2019).

Considering that BAG5 negatively regulates multiple cell protective mechanisms involving intracellular alpha-synuclein processing, we wanted to further investigate the molecular pathways in which BAG5 may function to advance our understanding of BAG5 as a potential modulator of synucleinopathies. We therefore used a mass spectroscopy screen to find potential BAG5 interacting proteins. Here we identify and validate a functional interaction between BAG5 and p62, a protein with important functions in the ALP (Gamerdinger et al., 2009) previously shown to protect against alpha-synuclein pathology (Tanji et al., 2015). We subsequently assess the effects of BAG5 and p62 on alpha-synuclein oligomer levels and find that BAG5 can enhance oligomer formation as well as regulate p62 levels and subcellular distribution.

## Materials and Methods

### Cell Culture

H4 and HEK293 cells were cultured in Dulbecco’s Modified Eagle Medium (DMEM, Gibco) supplemented with 10% fetal bovine serum (Gibco), 1% antibiotic/antimycotic (Gibco), and incubated at 37°C with 5% CO_2_. H4 cells were exclusively grown on cell+ plates (Sarstedt, Inc.).

### Generation of Stable Cell Lines

Wild-type H4 neuroglioma cells were stably transfected with GFP, GFP-BAG5, or GFP-BAG5_DARA_ plasmids using Lipofectamine 2000 (Thermo Fisher Scientific) according to the manufacturer’s protocol. The GFP-BAG5 and GFP-BAG5_DARA_ plasmids were originally developed by Kalia et al. (2004) by inserting BAG5 and BAG5_DARA_ into the pEGFP-C1 plasmid (Clontech, U55763), which contains an N-terminal GFP tag and a eukaryotic G418 resistance gene. 24 h post-transfection, cells were incubated in selection media containing 700 μg/mL G418 for 14 days. Cell colonies that reached a size of 100–200 cells were assessed for GFP-transgene incorporation using fluorescence microscopy. Those colonies stably expressing the transgene were transferred to a 96-well plates and propagated for further characterization of transgene expression.

### Immunoprecipitation of GFP-Fusion Proteins

H4 GFP, GFP-BAG5, and GFP-BAG5_DARA_ cell lines were plated in 10 cm plates. 24 h after plating the cells were washed with 5 mL Dulbecco’s phosphate buffered saline (PBS) without calcium or magnesium and lysed in radioimmunoprecipitation assay (RIPA) buffer composed of 50 mM Tris, 150 mM NaCl, 0.5% sodium deoxycholate, 1% Triton X-100 and protease inhibitor cocktail (cOmplete^TM^, Roche). For the immunoprecipitation, 1 mg of protein lysate from each cell line was combined with 25 μL of pre-washed GFP-trap bead slurry (Chromotek, gta-10) and rotated at 4°C for 2 h. Beads were subsequently washed three times with 1 mL of RIPA buffer, and protein samples were transported to the SPARC BioCentre Molecular Analysis, The Hospital for Sick Children, Toronto, ON, Canada for mass spectrometry analysis.

### Mass Spectrometry

Mass spectrometry was performed by SPARC BioCentre Molecular Analysis, The Hospital for Sick Children, Toronto, ON, Canada. Samples were prepared for analysis by suspending immunoprecipitation samples in 50 mM NH_4_HCO_3_. Dithiothreitol (DTT) was added to a final concentration of 10 mM and samples were heated at 60°C for 30 min. After the samples returned to room temperature, iodoacetamide was added to a final concentration of 10 mM and samples were incubated at room temperature in the dark for 15 min. Iodoacetamide was inactivated by adding DTT to a final concentration of 40 mM. Trypsin was then added to the sample to a protease:protein ratio of 1:50 (w/w) and digestion took place overnight at 37°C. Samples were analyzed on a linear ion trap-Orbitrap hybrid analyzer (LTQ-Orbitrap, Thermo Fisher Scientific, San Jose, CA, United States) outfitted with a nanospray source and EASY-nLC split-free nano-LC system (Thermo Fisher Scientific, San Jose, CA, United States). Lyophilized peptide mixtures were dissolved in 0.1% formic acid and loaded onto a 75 μm × 50 cm PepMax RSLC EASY-Spray column filled with 2 μm C18 beads (Thermo Fisher Scientific, San, Jose, CA, United States) at a constant pressure of 800 BAR. Peptides were eluted over 60 min at a rate of 250 nl/min using a 0–35% acetonitrile gradient in 0.1% formic acid.

Peptides were introduced by nanoelectrospray into an LTQ-Velos-Orbitrap Elite hybrid mass spectrometer (Thermo Fisher Scientific). The instrument method consisted of one MS full scan (400–1500 m/z) in the Orbitrap mass analyzer, an automatic gain control target of 1e6 with a maximum ion injection of 100 ms, one microscan, and a resolution of 240,000. Ten data-dependent MS/MS scans were performed in the linear ion trap using the ten most intense ions at 35% normalized collision energy. The MS and MS/MS scans were obtained in parallel fashion. In MS/MS mode automatic gain control targets were 30,000 with a maximum ion injection time of 50 ms. A minimum ion intensity of 1000 was required to trigger an MS/MS spectrum. Normalized Collision Energy was set at 35. The dynamic exclusion was applied using a maximum exclusion list of 500 with one repeat count with a repeat duration of 30 s and exclusion duration of 15 s.

Tandem mass spectra were extracted, charge state deconvoluted and deisotoped by Xcalibur version 2.2. All MS/MS samples were analyzed using PEAKS Studio [Bioinformatics Solutions, Inc., Waterloo, ON, Canada; version 8.0 (2016-06-21)] and X! Tandem [The GPM^1^; version CYCLONE (2010.12.01.1)]. Data was searched with a fragment ion mass tolerance of 0.60 Da and a parent ion tolerance of 10.0 PPM.

Scaffold (version Scaffold_4.8.1, Proteome Software, Inc., Portland, OR, United States) was used to validate MS/MS based peptide and protein identifications. Peptide identifications were accepted if they could be established at greater than 95.0% probability. Peptide Probabilities from X! Tandem were assigned by the Scaffold Local FDR algorithm. Peptide Probabilities from PEAKS Studio were assigned by the Peptide Prophet algorithm (Keller et al., 2002) with Scaffold delta-mass correction. Protein identifications were accepted if they could be established at greater than 95.0% probability and contained at least five identified peptides. Protein probabilities were assigned by the Protein Prophet algorithm (Nesvizhskii et al., 2003). Proteins that contained similar peptides and could not be differentiated based on MS/MS analysis alone were grouped to satisfy the principles of parsimony. The mass spectrometry proteomics data have been deposited to the ProteomeXchange Consortium via the PRIDE (Perez-Riverol et al., 2019) partner repository with the dataset identifier PXD019473 and 10.6019/PXD019473.

### GST-Pull-Down Assays

Full length p62-HA was a gift from Qing Zhong (Addgene plasmid #28027) and deletion constructs for: (1) “p62-N-HA” (aa1-102 including PB1 domain) and (2) “p62-C-HA” [aa103-440 including LC3 interacting region (LIR) and ubiquitin associated (UBA) domains] were generated using the Q5 Site-Directed Mutagenesis Kit (NEB) as per the manufacturer’s protocol.

GST, GST-BAG5, and GST-BAG5_DARA_ recombinant proteins were generated in *Escherichia coli* using pGEX, pDEST-15-BAG5, and pDEST-15-BAG5_DARA_ (Gateway cloning system, Thermo Fisher Scientific), respectively. Recombinant proteins were conjugated to Glutathione Sepharose 4B (GE Healthcare) beads by rotating recombinant protein with bead slurry overnight at 4°C in PBS.

H4 cells were transiently transfected with p62-HA, p62-N-HA, and p62-C-HA using Lipofectamine 2000 (Thermo Fisher Scientific) according to the manufacturer’s protocol and lysed with RIPA buffer. 500 μg of cell lysate was incubated with 10 μg of conjugated GST-fusion protein beads overnight at 4°C with rotation. Beads were subsequently washed three times with 1 mL RIPA buffer, and protein was recovered from the bead slurry by adding 50 μL SDS-PAGE sample buffer (with beta-mercaptoethanol) and heat denaturing the sample at 95°C for 10 min.

### Alpha-Synuclein Protein Complementation Assay

Alpha-synuclein luciferase constructs were generated as previously described (Outeiro et al., 2008; Kalia et al., 2011). syn-N and syn-C were transfected into HEK293 cells in 6-well plates using Lipofectamine 2000 (Thermo Fisher Scientific) as per the manufacturer’s protocol. 24 h post-transfection, cells were scraped in 600 μL cold PBS and 100 μL of cells were transferred in triplicate to an opaque flat-bottomed 96-well plate (Grenier). The other 300 μL of cells were saved for western blot analyses. The plate was then analyzed on a CLARIOstar plate-reader (BMG Labtech), which injected 100 μL of 40 μM coelenterazine into each well and shook the plate for 2 s prior to reading the bioluminescent signal. Coelenterazine (303-5) was obtained from NanoLight Technology.

### Western Blotting

H4 cells were lysed with RIPA buffer containing protease inhibitor cocktail (Roche). The triton X-100 soluble fraction was then separated from the insoluble pellet by centrifugation. Protein concentration was quantified using the DC (Bradford) protein assay (BioRad). For each condition, 20 μg of protein lysate was run on 4–15% acrylamide gels (BioRad) and subsequently transferred onto a polyvinylidine fluoride (PVDF) membrane. Blots were blocked with 5% skim milk in TBS + 0.01% Tween-20 (TBS-T) for 30 min prior to incubation with primary antibody for either 1 h at 21°C or overnight at 4°C. Western blot and immunostaining antibodies were purchased from the following suppliers: anti-alpha-synuclein (610786) and anti-p62/SQSTM1 (610833) were obtained from BD biosciences. Anti-actin (A2066) was obtained from Sigma-Aldrich. Anti-BAG5 (CSB-PA890743ESR1HU) was obtained from Cusabio. Anti-GFP (A11122) was obtained from Invitrogen. Anti-HA (11867423001) was obtained from Roche. Blots were subsequently washed three times in TBS-T for 10 min per wash, incubated in species specific secondary antibody for 1 h at 21°C, washed again, and then developed using ECL western blotting substrate (Pierce) and visualized on HyBlot CL autoradiographic film (Denville Scientific, Inc.). Band intensity quantification was performed on ImageJ software by inverting the Western blot image, measuring light intensity of the band and subtracting the light intensity of the background. Band intensity was subsequently normalized to the band intensity of actin in the corresponding well.

### Immunocytochemistry

Wild-type H4 cells were plated at 70–80% confluency in 24-well plates containing poly-D lysine treated glass cover slips. For knockdown experiments, cells were transfected with siRNAs either targeting BAG5 (siBAG5, Thermo Fisher Scientific 4392420) or non-targeting control (siNTC, Thermo Fisher Scientific 4390843) at the time of plating using Lipofectamine RNAiMAX (Thermo Fisher Scientific) as per the manufacturer’s protocol. 48 h after transfection, cells were washed once with PBS and treated with 4% paraformaldehyde (PFA) for 15 min at room temperature. The PFA was washed off with three sequential 5 min PBS washes and the cells were subsequently treated with 0.2% triton X-100 diluted in PBS for 15 min at room temperature. Cells were then washed another three times in PBS and blocked with 5% weight/volume bovine serum albumin (BSA) diluted in PBS for 45 min. BAG5 and p62 antibodies were diluted 1:1000 in 5% BSA/PBS and incubated with the cells overnight at 4°C with gentle rocking. Cells were washed in PBS, incubated in species specific Alexa Fluor 488 and 555 (Thermo Fisher Scientific A11034 and A21424, respectively), and washed again in PBS containing 4′,6-Diamidino-2-Phenylindole, Dihydrochloride (DAPI). Finally, the coverslips were mounted onto slides with fluorescent mounting media (Dako, S3023).

## Results

### Screen for BAG5 Interacting Proteins

**FIGURE 1:**
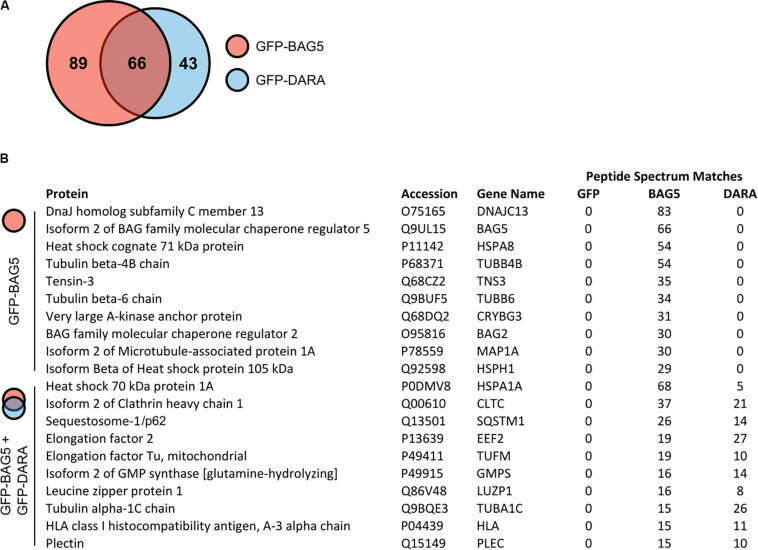
Mass spectrometry screen for GFP-bcl-2 associated athanogene 5 (BAG5) and GFP-BAG5_DARA_ interacting proteins. **(A)** Venn diagram illustrating the proteins identified in the screen for BAG5 interacting proteins in H4 cells. 198 proteins were identified in total, 89 were found to be in complex with GFP-BAG5 (red), 43 with GFP-BAG5_DARA_ (GFP-DARA, blue), and 66 with both. **(B)** List of the top 10 proteins identified to co-immunoprecipitate with GFP-BAG5 alone (top) and the top 10 proteins identified to co-immunoprecipitate with both GFP-BAG5 and GFP-BAG5_DARA_ (bottom).

To explore potential BAG5 interacting proteins, we created H4 cell lines stably expressing GFP-BAG5 or GFP as a negative control. The H4 neuroglioma cell line was chosen because it has been previously used to interrogate BAG5 function and PD-relevant molecular pathways, including alpha-synuclein aggregation (McLean et al., 2002, 2004; Klucken et al., 2006, 2012; Kalia et al., 2011; Danzer et al., 2012). We made an additional cell line stably expressing GFP-BAG5_DARA_, a BAG5 mutant we previously demonstrated to have limited ability to bind Hsp70 due to mutation of key aspartate and arginine residues in the BAG domain (Kalia et al., 2004, 2011). This GFP-BAG5_DARA_ cell line allowed us to identify those proteins that likely bind to BAG5 independently of Hsp70. We isolated *in vitro* protein complexes by immunoprecipitation (IP) with an anti-GFP antibody and screened for co-immunoprecipitated proteins using mass spectrometry (MS). This screen identified 198 BAG5 interacting proteins of which 89 bound to GFP-BAG5, 43 bound to GFP-BAG5_DARA_, and 66 bound to both (Figure 1A and Supplementary Table S1). The top 10 proteins identified in the BAG5 IP complexes, as well as those found in both the BAG5 and BAG5_DARA_ IP complexes are listed in Figure 1B. Hsp70 family members, such as HSPA8 and HSPA1A, were more abundant in the BAG5 IP condition relative to the GFP-BAG5_DARA_ IP condition, as gauged by total spectral counts, providing internal validation of our technique. We focused on the latter since they represent proteins that interact with BAG5 but likely do not require Hsp70 to mediate the interaction. We reasoned that these proteins would be less likely to form a complex with BAG5 through non-specific interactions with the protein binding domain of Hsp70 which binds misfolded or unfolded protein clients of Hsp70.

### BAG5 Interacts With p62

**FIGURE 2:**
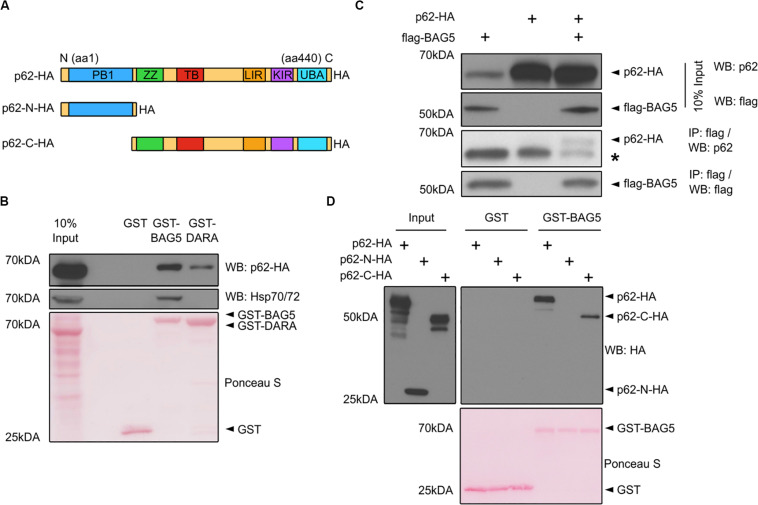
BAG5 interacts with p62. **(A)** Domain structure of p62 and the deletion constructs (p62-N-HA and p62-C-HA) generated to map its interaction with BAG5. PB1, Phox and Bem1p; ZZ, ZZ-type zinc finger; TB, TRAF6 binding; LIR, LC3 interacting region; KIR, keap-1 interacting region; UBA, ubiquitin-associated. All p62 constructs contained a C-terminal hemagglutinin (HA) tag. **(B)** Pull-down assays were performed with GST fusion proteins and lysates from H4 cells transfected with p62-HA to validate the interaction between BAG5 and p62. Proteins were probed with anti-p62 (upper) and anti-heat shock protein 70 (Hsp70; middle) antibodies. GST fusion proteins were stained with Ponceau S (bottom). Input was 10% of the total protein used for each condition in the pull-down assays. Molecular weight markers are indicated on the left. Results are representative of three independent experiments. **(C)** Immunoprecipitation (IP) of FLAG-BAG5 using lysates from H4 cells transfected with FLAG-BAG5 and/or p62-HA. Input was 10% of the total protein used for each IP (top two panels). Immunoprecipitants were sequentially probed with anti-HA and anti-FLAG antibodies (bottom two panels). * indicates the heavy chain of the IP antibody. Similar results were observed in three separate experiments. **(D)** Pull-down assays were performed with GST fusion proteins and lysates from H4 cells transfected with constructs shown in **(A)** to map the BAG5-p62 interaction. Input was 10% of the total protein used for each condition in the pull-down assays (left). Proteins were probed with anti-HA antibody (right). GST fusion proteins were stained with Ponceau S (bottom). Molecular weight markers are indicated on the left. Results are representative of three independent experiments.

p62 was one of the top 10 proteins identified in both the BAG5 and BAG5_DARA_ IP complexes (Figure 1B). p62 has important functions in autophagy whereas the function of BAG5 in autophagy is poorly understood and has only been indirectly investigated (Beilina et al., 2014). p62 is a multidomain protein (Figure 2A) containing a N-terminal Phox and Bem1p (PB1) domain which supports the capacity of p62 to self-associate and facilitates the formation of protein aggregates. The C-terminal LIR and UBA domains of p62 allow it to link ubiquitinated protein aggregates to the autophagy machinery for subsequent degradation (Bitto et al., 2014; Liu et al., 2017).

We first confirmed the interaction between BAG5 and p62 using GST pull-down assays in which recombinant GST-BAG5 or GST-BAG5_DARA_ was incubated with H4 cell lysates transfected with HA-tagged p62 (p62-HA). Both GST-BAG5 and GST-BAG5_DARA_, but not GST alone, pulled down p62-HA (Figure 2B). Consistent with our previous findings (Kalia et al., 2004, 2011) and our interactome analysis, Hsp70 was not pulled down by GST-BAG5_DARA_ or GST alone (Figure 2B), demonstrating that the BAG5-p62 interaction is not dependent on Hsp70. To confirm the interaction between p62 and BAG5 in a separate assay, we next performed IPs using lysates from H4 cells expressing p62-HA with FLAG-BAG5, and we found that p62-HA co-immunoprecipitated with FLAG-BAG5 (Figure 2C).

To determine which region of p62 mediates the p62-BAG5 interaction, we generated deletion constructs containing either the PB1 domain alone (p62-N-HA) or the LIR and UBA domains with the PB1 domain deleted (p62-C-HA) which we used in pull-down assays (Figure 2A). We found that GST-BAG5 pulled down p62-C-HA but not p62-N-HA (Figure 2D). Thus, using both pull-down and co-immunoprecipitation assays, we confirmed the BAG5-p62 interaction that was identified from our MS screen. Furthermore, we determined that the C-terminal region of p62, containing the UBA and LIR domains, is sufficient for binding to BAG5.

### BAG5 Regulates p62 Protein Levels

**FIGURE 3:**
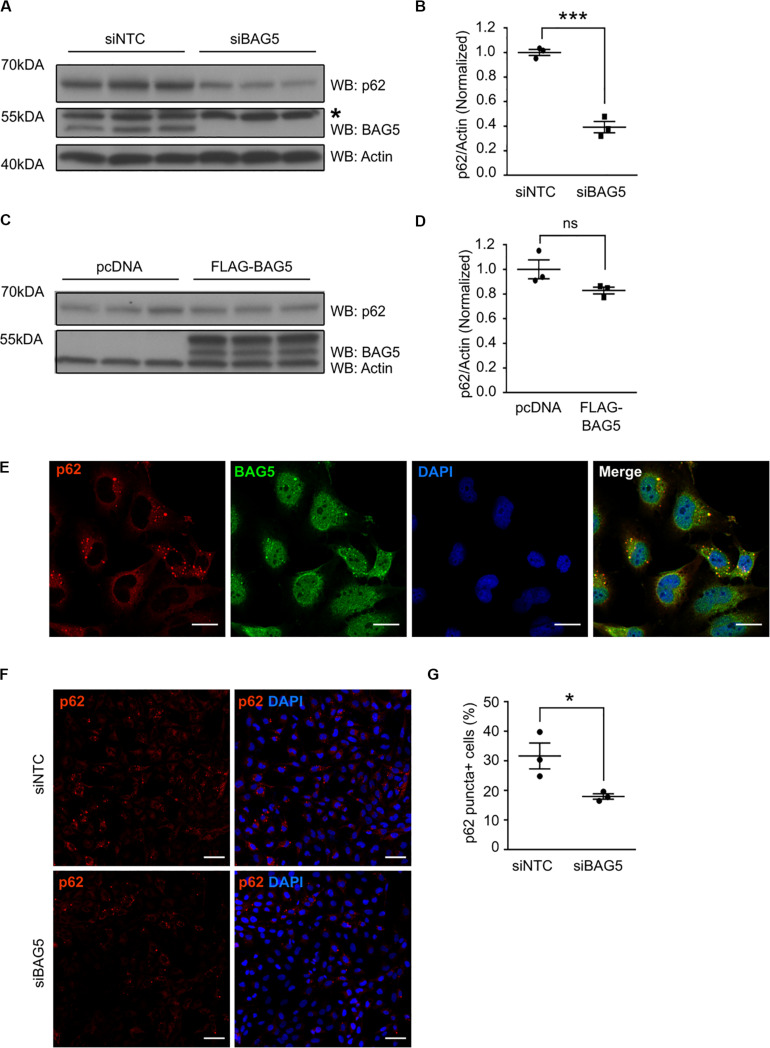
BAG5 regulates p62 protein levels. **(A)** Western blots of lysates from H4 cells treated with either siNTC or siBAG5. Proteins were sequentially probed with anti-p62, anti-BAG5, and anti-actin antibodies. Actin served as the loading control. * indicates a non-specific band. Three independent experiments are included on the blot. **(B)** Densitometric quantification of the change in endogenous p62 elicited by BAG5 KD from the immunoblots in **(A)**. p62 band intensity was normalized to actin and siNTC condition (***p < 0.001, independent samples *t*-test, *n* = 3). **(C)** Western blots of lysates from H4 cells transfected with either pcDNA control plasmid or FLAG-BAG5. Proteins were sequentially probed with anti-p62, anti-BAG5, and anti-actin antibodies. Actin served as the loading control. Three independent experiments are included on the blot. **(D)** Densitometric quantification of endogenous p62 from the immunoblots in **(C)**. p62 band intensity was normalized to actin and siNTC condition (ns, no significance, independent samples *t*-test, *n* = 3). **(E)** Endogenous BAG5 (green) and p62 (red) were visualized in H4 cells using immunofluorescent staining. Cell nuclei were visualized with 4’,6-Diamidino-2-Phenylindole (DAPI) (blue). BAG5 demonstrated nuclear and cytoplasmic staining, while p62 was largely excluded from the nucleus and clustered into small puncta in the perinuclear space. BAG5 co-localized with most, but not all, of these puncta (yellow). Scale bar represents 20 μm. Results are representative of three independent experiments. **(F)** Endogenous p62 (red), including p62 in perinuclear puncta, was visualized using immunofluorescent staining in H4 cells treated with siNTC or siBAG5. Cell nuclei were visualized with DAPI (blue). Scale bar represents 50 μm. Representative image from three independent experiments. **(G)** Quantification of the percentage of cells with visible perinuclear p62-containing puncta (p62 puncta+) using the immunofluorescence images pictured in **(F)**. A minimum of 200 cells were counted blinded. Data are representative of three independent experiments (*p < 0.05, independent samples *t*-test, *n* = 3). All graphs represent mean ± SEM.

To investigate the functional consequences of the interaction between BAG5 and p62, we investigated the effects of BAG5 on p62 protein levels. Homeostatic levels of p62 have been shown to be important for its function in protein aggregation and autophagy (Komatsu et al., 2007; Gamerdinger et al., 2009). BAG3 was previously shown to associate with p62 and support its stability, which promotes autophagy-mediated proteostasis in aging cells (Gamerdinger et al., 2009). In H4 cells, we found that targeted knockdown (KD) of BAG5 using siRNA reduced endogenous p62 protein levels by 60.8% (SEM = 4.6%) relative to the non-targeting control siRNA (*p* < 0.001, Figures 3A,B), suggesting that, similar to BAG3, BAG5 may support p62 stability. In contrast, overexpression of FLAG-BAG5 did not have a significant effect on endogenous p62 protein levels (Figures 3C,D).

p62 is known to associate with and coordinate the formation of protein aggregates that are destined for autophagic degradation (Bjørkøy et al., 2005). Using immunofluorescence to determine the cellular localization of both BAG5 and p62, we found that p62 was largely excluded from the nucleus but substantially enriched in perinuclear puncta (Figure 3E), similar to previously described subcellular localization of p62 protein aggregates (Watanabe and Tanaka, 2011). BAG5 exhibited a more diffuse distribution, including in the cytoplasm, perinuclear region, and nucleus as we have shown previously (Kalia et al., 2004). Notably, BAG5 was also observed in the perinuclear aggregates and co-localized with p62 (Figure 3E). Given that BAG5 KD reduced levels of soluble p62, we next tested the effect of BAG5 KD on the subcellular distribution of p62. We found that BAG5 KD reduced the proportion of H4 cells demonstrating perinuclear p62 puncta from 31.6% (SEM = 4.4%) to 17.9% (SEM = 0.9%, *p* < 0.05, Figures 3F,G), suggesting that BAG5 supports either the formation or stability of these structures. Together these results indicate that BAG5 appears to be important for maintaining levels of triton-soluble p62 as well as p62-associated protein aggregates, which may be through stabilization of p62.

### BAG5 Regulates Levels of Alpha-Synuclein Oligomers and p62

**FIGURE 4:**
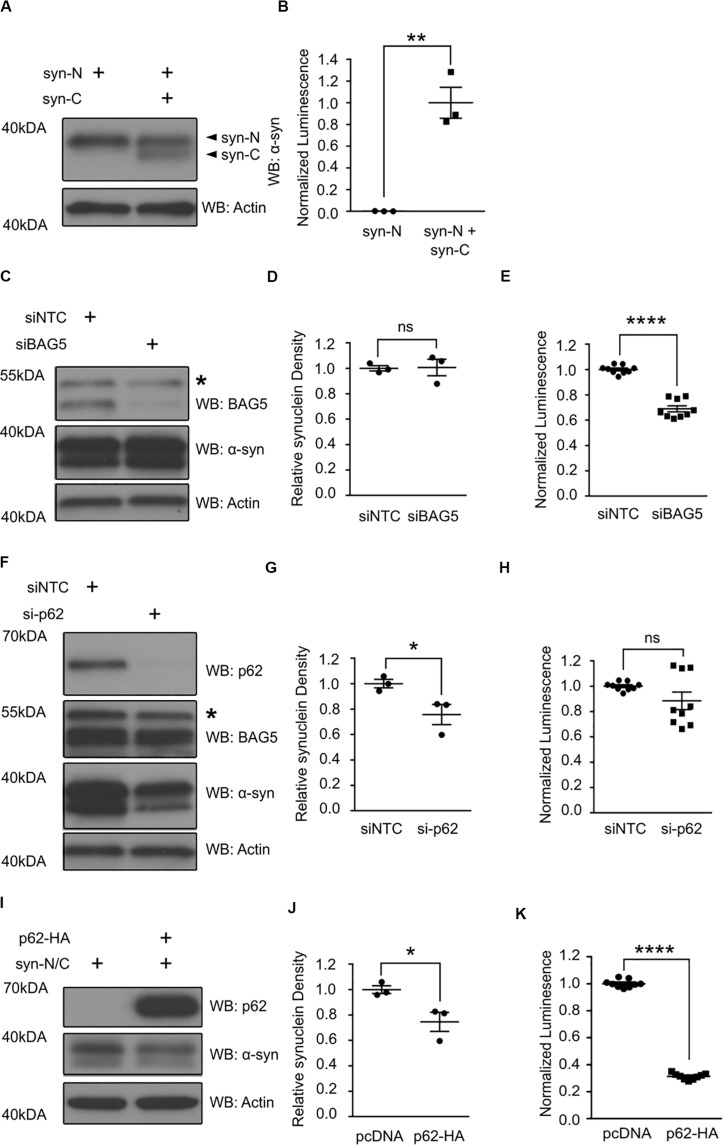
p62 and BAG5 modulate alpha-synuclein oligomerization. **(A)** Western blot illustrating the presence of the two alpha-synuclein constructs fused to either the N-terminus (syn-N) or the C-terminus (syn-C) of *Gaussia princeps* luciferase for the luciferase protein complementation assay (PCA) results presented in **(B)**. **(B)** Luminescent signal is only generated when both syn-N and syn-C are present. (**p < 0.01, independent samples *t*-test, *n* = 3). **(C)** Western blot illustrating the levels of BAG5 and syn-N/syn-C for the PCA presented in **(E)**. Representative of three independent studies. * indicates a non-specific band. **(D)** Densitometric quantification of alpha-synuclein protein in **(C)** [not significant (ns), independent samples *t*-test, *n* = 3]. **(E)** PCA illustrating the effect of small interfering RNA (siRNA)-mediated BAG5 knockdown (KD) on alpha-synuclein oligomer formation. (****p < 0.0001, independent samples *t*-test, *n* = 3). **(F)** Western blot illustrating the levels of p62, BAG5, and syn-N/syn-C for the PCA presented in **(H)**. Representative of three independent studies. * indicates a non-specific band. **(G)** Densitometric quantification of alpha-synuclein protein in **(F)** (*p < 0.05, independent samples *t*-test, *n* = 3). **(H)** PCA illustrating the effect of siRNA-mediated p62 KD on alpha-synuclein oligomer formation. [not significant (ns), independent samples *t*-test, *n* = 3] **(I)** Western blot illustrating the levels of p62 and syn-N/syn-C for the PCA presented in **(K)**. Representative of three independent studies. **(J)** Densitometric quantification of alpha-synuclein protein in **(I)** (*p < 0.05, independent samples *t*-test, *n* = 3). **(K)** PCA illustrating the effect of p62 overexpression on alpha-synuclein oligomer formation. (****p < 0.0001, independent samples *t*-test, *n* = 3). All graphs represent mean ± SEM.

The interaction of BAG5 with the region of p62 containing the UBA and LIR domains suggests that the BAG5-p62 interaction is relevant to protein aggregation and/or degradation. BAG5 is known to indirectly enhance alpha-synuclein oligomer formation, whereas p62 is known to facilitate alpha-synuclein aggregate degradation (Kalia et al., 2011; Watanabe et al., 2012; Tanji et al., 2015). Thus, we tested how BAG5 and p62 may modulate alpha-synuclein oligomers using a previously characterized luciferase reporter protein complementation assay (PCA) that allows for the analysis of alpha-synuclein oligomer levels in living HEK293 cells (Outeiro et al., 2008; Kalia et al., 2011). This model makes use of two alpha-synuclein constructs that each contains full-length alpha-synuclein fused to either the N-terminal or C-terminal half of *Gaussia princeps* luciferase (termed syn-N and syn-C, respectively). When alpha-synuclein oligomers form, the luciferase halves come in close proximity to reconstitute a fully active luciferase that can generate a measurable bioluminescent signal (Figures 4A,B). PCA bioluminescence is therefore a surrogate measure of alpha-synuclein oligomer levels as we and others have previously shown (Kalia et al., 2011).

Using this luciferase PCA to measure alpha-synuclein oligomers, we found that siRNA-mediated KD of BAG5 reduced the luciferase signal by 31.1% (SEM = 2.4%) relative to the non-targeting control siRNA (*p* < 0.0001, Figure 4E), without lowering the levels of soluble alpha-synuclein (Figures 4C,D). BAG5 KD also resulted in a substantial reduction in endogenous p62 (Figures 3A,B), suggesting a potential mechanism by which BAG5 modifies alpha-synuclein oligomer levels. p62 knockout has previously been shown to reduce the formation of neuronal protein aggregates (Komatsu et al., 2007) and thus we hypothesized that BAG5 KD decreases alpha-synuclein oligomers by reducing p62 levels. To directly test whether reduction in p62 levels is associated with decreased alpha-synuclein oligomers, we performed targeted KD of p62 with siRNA. We found that siRNA-mediated p62 KD had no significant effect on luciferase signal in the PCA (Figure 4H), despite reducing the levels of total soluble alpha-synuclein (Figures 4F,G). We also examined the effects of enhancing exogenous p62 levels on luciferase PCA. Overexpression of p62 significantly reduced levels of soluble alpha-synuclein (*p* < 0.04, Figures 4I,J) as well as luciferase signal by 68.8% (SEM = 0.8%) relative to pcDNA control (*p* < 0.0001, Figure 4K). Taken together, these findings demonstrate that p62 overexpression reduces alpha-synuclein oligomer levels and that the effect of BAG5 on alpha-synuclein oligomers is not mediated through a reduction of p62 levels but through an alternative mechanism.

## Discussion

Using a mass spectrometry-based screen for BAG5 interacting proteins, we found that BAG5 interacts with a rich network of chaperones as well as numerous proteins that function within the UPS and ALP. A novel Hsp70-independent interaction between BAG5 and p62 was identified and verified. These results suggest that BAG5 can interact with the ALP in an Hsp70-independent manner and may therefore serve as a molecular bridge between the chaperone network and the ALP. BAG5 has previously been implicated in autophagy as it forms a complex with the PD-relevant proteins LRRK2, Rab7L1, and cyclin-G-associated kinase (GAK) to promote turnover of the *trans*-Golgi network (TGN), an activity that impacts ALP function (Beilina et al., 2014). Interestingly, unlike the interaction between BAG5 and p62, the association of BAG5 with this complex was at least partially facilitated by Hsp70 (Beilina et al., 2014). More recently, we have demonstrated that BAG5 also modulates parkin-dependent mitophagy suggesting that this co-chaperone may have a more general role in regulating these processes (De Snoo et al., 2019). Further investigation will be required to understand the mechanisms by which this occurs and the role that other BAG proteins may play in these complex intracellular processes required to maintain proteostasis. In addition, while the H4 neuroglioma cell line used in this study is an accepted *in vitro* model for the interrogation of PD-relevant molecular pathways, the function of the BAG5-p62 interaction merits further investigation *in vivo*.

p62 is known to regulate the aggregation and degradation of proteins associated with neurodegenerative disease including alpha-synuclein as well as tau (which aggregates in Alzheimer’s disease), and huntingtin (which aggregates in Huntington’s disease) (Babu et al., 2005; Watanabe et al., 2012; Kurosawa et al., 2015; Tanji et al., 2015). Moreover, p62 is often co-localized with the protein aggregates observed in these neurodegenerative proteinopathies (Zatloukal et al., 2002). Therefore, the observations that BAG5 interacts with p62, promotes soluble p62 levels, and co-localizes with and supports the stability of p62 perinuclear aggresomes, suggest that this interaction may have important implications in disease-associated disturbances of proteostasis.

Using the luciferase PCA, we found that BAG5 enhanced both alpha-synuclein oligomer and p62 protein levels. Considering that p62 has previously been shown to support the formation of neuronal protein aggregates (Komatsu et al., 2007), we hypothesized that the effect of BAG5 on alpha-synuclein oligomers was mediated by its effect on p62 protein levels. However, targeted p62 KD on its own was not sufficient to reduce alpha-synuclein oligomers in our model although there was a significant reduction in alpha-synuclein levels. Therefore, it is likely that the effect of BAG5 on alpha-synuclein oligomerization in this study was mediated through alternative p62-independent pathways. For example, BAG5 is known to inhibit the E3 ligase activity of CHIP, which, in turn, inhibits CHIP-mediated proteasomal degradation of alpha-synuclein and enhances alpha-synuclein oligomerization (Kalia et al., 2011). This could partially explain how BAG5 can enhance alpha-synuclein oligomerization in a p62-independent manner. Moreover, the numerous proteins involved in the UPS and ALP that were identified as putative BAG5 interactors in our proteomic screen indicates that further research is required to dissect the complex molecular mechanisms that mediate the effect of BAG5 on alpha-synuclein oligomerization and degradation.

Given the well-characterized role of p62 in the formation and subsequent degradation of protein aggregates, it was surprising that p62 KD alone did not reduce alpha-synuclein oligomers. However, only two previous studies have directly assessed the effect of p62 on alpha-synuclein aggregation and suggested that p62 supports the lysosomal degradation of larger alpha-synuclein aggregates (Watanabe et al., 2012; Tanji et al., 2015). Therefore, it is possible that p62 has more of an impact on larger alpha-synuclein aggregates that may not be directly measured by the PCA, particularly fibrils. Consequently, the effect of BAG5 on p62 may only be relevant to these other forms of aggregated alpha-synuclein and future studies will benefit from assessing the functional relevance of the BAG5-p62 interaction on the aggregation and degradation of these larger alpha-synuclein aggregates. Another possible explanation of this finding is that other autophagy adaptor proteins, such as NBR1, compensate for the loss of p62 by promoting the oligomerization of alpha-synuclein. Indeed, NBR1 has been previously shown to co-localize with alpha-synuclein aggregates in PD subjects and support alpha-synuclein aggregation *in vitro* (Odagiri et al., 2012). Interestingly, overexpression of p62 reduced both oligomeric forms and total soluble alpha-synuclein. This effect may be through p62-mediated aggregation of ubiquitinated proteins, followed by subsequent targeting of aggregates for degradation via the ALP (Komatsu et al., 2007; Pankiv et al., 2007).

Considering that BAG5 functionally interacts with LRRK2 to promote turnover of the TGN (Beilina et al., 2014) and inhibits parkin-mediated mitophagy (De Snoo et al., 2019), it may also be possible that BAG5 has a downstream inhibitory effect on autophagic flux, which, in turn, slows the degradation of p62 and alpha-synuclein via the ALP and promotes the accumulation of p62 and alpha-synuclein aggregates (Wu et al., 2015). This notion is supported by the reduction in alpha-synuclein oligomers, soluble p62 protein levels, and perinuclear p62 aggregates following BAG5 KD. However, BAG5 KD did not have a significant effect on soluble alpha-synuclein protein levels. It is alternatively possible that BAG5 promotes the homo- and hetero-oligomerization of p62 or its capacity to interact with ubiquitinated protein aggregates (Liu et al., 2016), in turn explaining the reduction of p62 positive protein aggregates following BAG5 KD. Importantly, however, the complex interplay between p62, BAG5, alpha-synuclein, the UPS, and the ALP is likely not captured by any of these mechanisms in isolation. Indeed, p62 acts as a molecular bridge between the UPS and ALP and facilitates the upregulation of ALP activity following UPS inhibition (Lim et al., 2015; Liu et al., 2016), BAG5 has modulatory effects on the ALP and UPS via its interaction with CHIP (Kalia et al., 2011), LRRK2 (Beilina et al., 2014), and parkin (Kalia et al., 2004), and misfolded alpha-synuclein is known to induce dysfunction in both the UPS and ALP (Winslow et al., 2010; McKinnon et al., 2020). Furthermore, while the interaction between BAG5 and p62 does not appear to require Hsp70, it is possible that both Hsp70-dependent and independent pathways are relevant to the function of a BAG5-p62 interaction given the known co-chaperone activity of BAG5 (Kalia et al., 2004). Therefore, further work will be needed to understand the functional impact of the BAG5-p62 interaction on processes such as autophagic flux and UPS function in order to untangle the complexities of this interaction in the context of alpha-synuclein aggregation. Previous investigations of BAG5 function have demonstrated its interaction with both Hsp70 and members of the UPS (Kalia et al., 2004, 2011). Our results demonstrate that BAG5 also associates with the ALP in an Hsp70-independent manner via its interaction with p62. This extends the role of BAG5 in proteostasis pathways and suggests that understanding the relationship between BAG5 and autophagy is an important avenue of future investigation. We also demonstrate that BAG5 promotes alpha-synuclein oligomer formation through a p62-independent mechanism. This suggests a more complicated mechanistic relationship of how p62 may modulate alpha-synuclein processing. Taken together, BAG5 appears to be a molecular hub that facilitates a functional association between multiple cellular processes including the chaperone network, apoptosis, and protein degradation and may serve as a potential modulator of alpha-synuclein oligomerization.

## Data Availability Statement

The mass spectrometry proteomics data have been deposited to the ProteomeXchange Consortium via the PRIDE (Perez-Riverol et al., 2019) partner repository with the dataset identifier PXD019473 and 10.6019/PXD019473.

## Author Contributions

EF, LK, and SK designed the experiments. EF, RE, LK, and SK wrote the manuscript. EF, YZ, MD, DO’H, VA, HC, SN, and KC performed the experiments. All authors contributed to the article and approved the submitted version.

## Conflict of Interest

The authors declare that the research was conducted in the absence of any commercial or financial relationships that could be construed as a potential conflict of interest.

## Footnotes

1 https://thegpm.org/

## Abbreviations

ALP: autophagy lysosome pathway
BAG: bcl-2 associated athanogene
GST: glutathione S-transferase
Hsp: heat shock protein
IP: immunoprecipitation
KD: knockdown
LRRK2: leucine-rich repeat kinase 2
MS: mass spectrometry
PCA: protein complementation assay
PINK1: PTEN-induced putative kinase 1
siRNA: small interfering RNA
UPS: ubiquitin–proteasome system.
